# Screening and analysis of differentially expressed circRNAs and miRNAs in chronic diabetic extremity wounds

**DOI:** 10.3389/fsurg.2022.1007312

**Published:** 2022-11-10

**Authors:** Xiaoliang Li, Dan Lv, Jiangfan Xie, Xiangyang Ye, Chengde Xia, Dewu Liu

**Affiliations:** ^1^ Medical Center of Burn plastic and wound repair, The First Affiliated Hospital of Nanchang University, Nanchang, China; ^2^ Department of Burns, Zhengzhou First People’s Hospital, Zhengzhou, China

**Keywords:** diabetes mellitus, chronic wound and burn healing, high-Throughput nucleotide sequencing, circRNA, miRNA

## Abstract

Increasing studies have shown that circular RNAs (circRNAs) and microRNAs (miRNAs) are related to the development of endocrine and metabolic diseases. However, there are few reports on the expression of circRNAs and miRNAs and their related co-expression and the expression of competitive endogenous RNA (ceRNA) in diabetic chronic refractory wounds. In this study, we compared the differential expression of circRNAs and miRNAs in diabetes chronic refractory wounds and normal skin tissues by high-throughput gene sequencing, and screened the differentially expressed circRNAs and miRNAs. Five abnormally expressed circRNAs and seven abnormally expressed miRNAs were detected by reverse transcription quantitative polymerase chain reaction PCR (RT-qPCR)to verify the results of RNA sequencing. We applied gene ontology (GO) to enrich and analyze dysregulated genes and elucidated their main functions *via* the Kyoto encyclopedia of genes and genomes analysis (KEGG). We constructed coding noncoding gene co-expression networks and ceRNA networks based on significantly abnormally expressed genes. According to the results of coding noncoding gene co-expression network analysis, hsa_circRNA_104175, hsa_circRNA_ 001588, hsa_circRNA_104330, hsa_circRNA_ 100141, hsa_circRNA_103107, and hsa_ circRNA_102044 may be involved in the regulation of the chronic intractable wound healing process in diabetes mellitus. This is particularly true in the regulation of vascular smooth muscle contraction-related pathways and the actin cytoskeleton, which affect the healing of chronic intractable wounds in diabetes. MiR-223-5p, miR-514a-3p, miR-205-5p, and miR-203-3p, which each have a targeting relationship with the above circRNAs, regulate the metabolism of nitrogen compounds in wound tissue by regulating NOD-like receptor signaling pathways, signaling pathways regulating the pluripotency of stem cells, microRNAs in cancer, and ECM-receptor interaction. This study showed circRNAs, miRNAs, and their network are associated with the development of chronic intractable wounds in diabetes, and our research identified the goals for new molecular biomarkers and gene therapy.

## Introduction

The formation of chronic intractable wounds is a common, high incidence complication of diabetes that can be a burden and inconvenience for recipients (1, 2). Although many studies have been carried out on chronic intractable wound disease and pathogenicity (3, 4), few studies have focussed on the gene regulation of chronic intractable diabetes mellitus. In recent years, there has been increasing evidence that non-coding RNAs (ncRNAs), including microRNAs (miRNAs) and circular RNA (circRNAs), regulate gene expression at the transcription and translation levels and play a vital role in the formation of chronic intractable wounds in diabetic meltlets (5, 6).

CircRNAs belong to endogenous non-coding RNA. They widely exist in eukaryotic cells and have a covalent ring structure. They are produced by selective or reverse splicing of RNA and consist of highly conserved exons and/or introns. CircRNAs are involved in the development and progression of various diseases (7). CircRNAs influences miRNAs activity and expression of downstream target genes through competitive coupling of miRNA sponge (8, 9). For example, circZNF91 includes 24 miR-23b-3p targets known to play an important role in keratinocyte differentiation (10). This indicates that circRNAs can bind to miRNAs and affect the biological pathway of miRNAs. Another example is that circRNA_100782 as a miR-124 sponge can regulate BxPC3 cell proliferation through the IL6-STAT3 pathway (11). Since circRNAs are stable molecule against exonucleases, it can be used as a biomarker in clinical samples. However, at present, the function of circRNAs in chronic refractory wounds of diabetes is rarely reported.

MiRNAs are endogenous non coding RNAs raging in size from 18 to 25 nucleotides. They inhibit transcription or promote mRNA degradation, by combining with th 3′ untranslated region (UTR) of the target mRNA. It regulates gene expression at the post transcriptional level and is closely related to the occurrence and development of many diseases. They regulate genes involved in transforming growth factor (TGF)-β including signal transduction, ECM deposition, epithelial mesenchymal transformation, and fibroblast proliferation and differention (12). Abnormal expression of miRNAs can affect the healing of diabetic chronic refractory wounds through various ways (13). For example, miRNA-203 promotes wound healing by promoting 52% keratinocyte migration, and miRNA-210 promotes wound healing by promoting 52% keratinocyte proliferation (14). However, miRNA-99 and miRNA-200 families can inhibit keratinocyte cell migration and delay wound healing (15).

A new strategy for wound healing research involves exploring noncoding RNA. CircRNAs act as competitive endogenous RNAs (ceRNAs), which are regulated by competing for the same miRNA response element (MRE) (16, 17). However, the potential role of cirRNAs and its target RNA in chronic intractable wounds in human remains unexplored. In this research, the expression levels of circRNAs and miRNAs in chronic refractory wounds of diabetes were detected by high-throughput gene sequencing. The co-expression and ceRNA network of these non-regulatory RNAs were analyzed, and the function of circRNAs and miRNAs were predicted.

## Materials and methods

### Patients and samples

Our experimental research was designed according to the basic principles and concepts of the Declaration of Helsinki, and the study does not violate the relevant principles and concepts. Our analytical study was initiated after obtaining the approval and consent of the Ethics Committee of Zhengzhou First People’s Hospital (Zhengzhou, China). All subjects were tested and divided into groups after obtaining informed consent. From May 2018 to October 2021, we collected a total of 8 specimens from chronic diabetic wounds and adjacent normal skin tissues in Zhengzhou First People’s Hospital (5 men and 3 women, age 45–58 years). All eight patients underwent surgery without drug treatment or radiotherapy before operation. Specimens were obtained from patients through sterile surgical procedures in a sterile operating room, where epidermis and subcutaneous tissue were removed under sterile conditions and stored in liquid nitrogen.

### RNA isolation and quality control

According to the instructions and its associated company’s operating instructions, TRIzol (Invitrogen, Thermo Fisher Scientific, Inc., Waltham, MA, United States) was used to isolate the whole RNA from diabetic chronic intractable wounds and normal skin tissues. Then, RNA from all sterile samples was washed in RNeasy (Qiagen, Valencia, CA, United States) and quantitatively analyzed using an Agilent 2100 Bioanalytical Unit (Agilent Technologies, Santa Clara, CA, United States) after washing. We assessed RNA integrity and chromosomal DNA contamination by denaturing agarose gel electrophoresis.

### Library preparation for RNA sequencing (RNA SEQ)

circRNA Arraystar was applied to screen candidate circRNAs and miRNA was identified using RNA-seq. We applied 5′-phosphate-dependent exonuclease [GGeneRead rRNA Depletion Kit (Qiagen, Germany)] for depletion of ribosomal RNA and ribonuclease R.Rayscript for depletion of linear RNA. Finally, a cDNA Synthesis kit (Genaey, gk8030) was used for reverse transcription. Synthesis of cDNA and enrichment of cDNA templates was performed using RNA fragments of the first and second sequences. Before sequencing, we employed an Agilent 2,100 Bioanalyzer (Agilent Technology Co., Ltd.) to assess the quality of the library. To determine total concentration, we used a Fluorometer from Qubit 12.0 (Thermo Fisher Scientific, Inc.). The libraries were sequenced on the Illumina NextSeq 500 platform (Illumana, Inc.), which was constructed and sequenced by Shanghai Personal Biotechnology Co., Ltd. (www.personalio.CN/; Shanghai, China).

### Data sorting and analysis

The raw data reads were quality controlled and monitored, and the remaining “clean reads” were finally mapped to the Ensembl database (www.ensembl.Org/) using Tophat version 2 (ccb.jhu.edu/software/Tophat/index.shtml). The number of reads per 1,000 bases per gene per million reads was calculated and used for the analysis of fold change. The differentially expressed genes (DEGs) were detected using DEGseq software (version 1.18.0). When the folding change >2, *p*-value <0.05, and false discovery rate (FDR) < 0.05, the gene expressed was considered to be statistically significantly different, which was the standard for reducing differential expression. The pheatmap software package was used to analyze the differentially expressed genes in each group.

The gene function annotation in this experiment included Ensembl ID, chromosome distribution information of each position, gene classification [GO, KEGG, orthodontics (KO)], enzyme classification, and gene name [human genome variation society symbol, National Biotechnology Information Center (NCBI) gene ID, UniProtKB ID], KEGG, orthodontics (KO), and enzyme classification]. The GO Consortium (www.geneontology.org/) was used to analyze the richness of GO functions.

### Validation of reverse transcription quantitative polymerase chain reaction PCR (RT-qPCR)

In this experiment, we used RT-qPCR technology to validate the RNA-seq data. Furthermore, we extracted RNA using TRIzolTM reagent (Beijing Solarbio Science & Technology Co., Ltd., Beijing, China), and reverse-transcribed the experimental target RNA using the Rayscript cDNA synthesis kit (Catalog No. gk8030; Shanghai General Biotechnology Co., Ltd., Shanghai, China). Generate cDNA. We also analyzed the sequence of the cDNA by RT-qPCR using power qPCR master mix (Catalog No. gk8020; Shanghai General Biotechnology Co., Ltd.). NCBI Primer BLAST (www.ncbi. NLM. NIH. Gov/tools/primer blast/) and OLIGO7.37 software were used to check and design primers. The software was constructed from the corresponding human RNA sequences in GenBank (www.ncbi.NLM.NIH.Gov/GenBank/). U6 and GAPDH was used as the control gene for normalizing the cDNA load difference. Data were statistically analyzed using Student’s *t*-test. *P* < 0.05 suggested statistical significance. The data results obtained from the index detection are expressed as mean ± standard deviation and analyzed using SPSS 19.0. The experiments were repeated three times.

### Co-expression network and ceRNA network analysis

The circRNAs and miRNAs expression levels were statistically analysed, and all meaningful associations were elucidated. In this experiment, we used Cytoscape software to search and find circRNAs and miRNAs sequences to find their potential miRNA response elements. The binding sites on circRNAs and miRNAs were used to predict circRNA-miRNA interactions. miRanda (www.microrna.Org/), Targetscan (www.Targetscan.Org/) and psRobot (omicslab.Genetics.Ac.cn/psRobot/) were used to predict the relationship between circRNAs, miRNAs and mRNAs.

## Results

### Different expression profiles of circRNAs and miRNAs determined by RNA-seq

**Figure 1:**
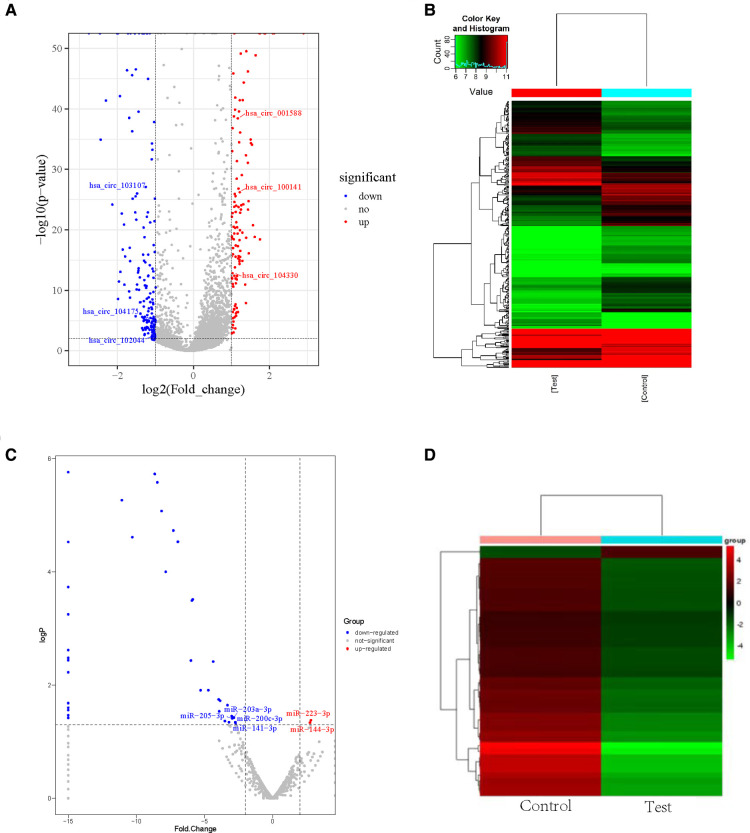
Differential cirRNAs and miRNAs expression profiles determined by RNA-seq. (**A**) Volcano plot of differential expression of circRNAs in wound tissues of diabetic patients. X-axis: log2 (FC); Y axis:—log10 (*p*-value); X-axis parallel line: *p* = 0.05; Y-axis parallel line: FC = 2; Red region: differential genes with *p* < 0.05 and FC ≥ 2; Blue region: differential genes with *p* < 0.05 and FC ≤ 0.5. (**B**) Differential expression of circRNA type heat picture in wound tissues of diabetic patients. The abscissa is the sample name, and the ordinate is the probe number. Black indicates that the gene expression level has not changed, red indicates that the expression level increases, and green indicates that the expression level decreases. The brightness of the color represents the degree of increase or decrease in gene expression. (**C**) The volcano plot visualizes the expression of miRNAs between chronic diabetic wound tissues and adjacent normal skin tissues (NC: normal tissue, Case: wound tissue) X axis: Log2 (FC); Y axis:—log10 (*p-*value); X-axis parallel line: *p* = 0.05; Y-axis parallel line: FC = 2; Red region: differential genes with *p* < 0.05 and FC ≥ 2; Blue region: differential genes with *p* < 0.05 and FC ≤ 0.5. D. Differential expression of miRNA type heat picture in wound tissue of diabetic patients. The abscissa is the sample name and the ordinate is the probe number. Black indicates that the gene expression level has not changed, red indicates that the expression level increases, and green indicates that the expression level decreases. The brightness of the color represents the degree of increase or decrease in gene expression.

We used RNA-seq to detect thousands of transcripts between diabetic tissues and normal tissues. We detected 356 diiferentially expressed circRNAs and 42 miRNAs (|fold change| > 2.0, *p* < 0.05) (Tables 1A–D). The volcano map of differential circRNAs expression was shown in Figure 1A, and the cluster analysis heat map results were shown in Figure 1B. Tables 1A, 1B enumerated the large differences genes. Among 42 differentially expressed miRNAs, 2 were upregulated, and 40 were downregulated. The volcano map results of differentially expressed miRNAs were shown in Figure 1C. The cluster analysis heat map results were shown in Figure 1D. The large difference in gene expression was listed in Tables 1C, 1D. We found that 157 circRNAs were upregulated and 199 were downregulated. There were large differences in gene expression between wound tissues and normal tissues in diabetic patients.

**Table 1A:** 20 circRNAs significantly upregulated in wound of diabetic patients.

circRNA	GeneSymbol	Fold Change	Regulation	miRNA Binding Sites
hsa_circRNA_089763	JA760600	9.7546075	up	hsa-miR-6803-3p
hsa_circRNA_089762	JA760602	7.4405905	up	hsa-miR-554
hsa_circRNA_402294	ACTR2	6.5318722	up	hsa-miR-1273g-5p
hsa_circRNA_004662	SOD2	4.864772	up	hsa-miR-4520-2-3p
hsa_circRNA_000926	ACTR2	4.8058478	up	hsa-miR-619-5p
hsa_circRNA_102747	ACTR2	4.6282329	up	hsa-miR-21-3p
hsa_circRNA_008882	MTND5	4.3567929	up	hsa-miR-4739
hsa_circRNA_101213	RAN	4.2319962	up	hsa-miR-431-3p
hsa_circRNA_104193	AHI1	3.7048804	up	hsa-miR-335-3p
hsa_circRNA_062683	TPST2	3.6999986	up	hsa-miR-3065-3p
hsa_circRNA_089761	JA760602	3.6480379	up	hsa-miR-3529-3p
hsa_circRNA_103188	TPST2	3.6298064	up	hsa-miR-593-5p
hsa_circRNA_034642	VPS18	3.4952925	up	hsa-miR-6836-5p
hsa_circRNA_101675	UBE2I	3.464019	up	hsa-miR-203a-5p
hsa_circRNA_104740	HAUS6	3.3752168	up	hsa-miR-186-5p
hsa_circRNA_100926	PICALM	2.7237195	up	hsa-miR-429
hsa_circRNA_100141	PTP4A2	2.6911287	up	hsa-miR-203a-3p
hsa_circRNA_001588	HIST1H4E	2.3312326	up	hsa-miR-514a-3p
hsa_circRNA_100927	PICALM	2.2724638	up	hsa-miR-429
hsa_circRNA_104330	CCDC126	2.1463146	up	hsa-miR-514a-3p

**Table 1B:** 20 circRNAs significantly downregulated in wound of diabetic patients.

circRNA	GeneSymbol	Fold change	Regulation	miRNA binding sites
hsa_circRNA_001226	MYH9	−23.8970437	down	hsa-miR-637
hsa_circRNA_051907	RPS11	−6.7989804	down	hsa-miR-4290
hsa_circRNA_406821	ARMC2	−6.1958784	down	hsa-miR-6876-5p
hsa_circRNA_405296	TUBGCP5	−5.7804753	down	hsa-miR-619-5p
hsa_circRNA_405769	C19orf55	−5.5502626	down	hsa-miR-216a-3p
hsa_circRNA_406246	SLC4A7	−5.4800209	down	hsa-miR-6888-5p
hsa_circRNA_406829	CDK19	−5.4668249	down	hsa-miR-3662
hsa_circRNA_061260	BAGE4	−5.4239204	down	hsa-miR-4720-5p
hsa_circRNA_405500	PKD1L2	−5.2735366	down	hsa-miR-3936
hsa_circRNA_000367	SIAE	−4.9734661	down	hsa-miR-331-3p
hsa_circRNA_101216	ANKLE2	−4.9678426	down	hsa-miR-204-3p
hsa_circRNA_400294	COL11A1	−4.4281753	down	hsa-miR-4668-3p
hsa_circRNA_103546	LPP	−4.0634159	down	hsa-miR-216a-5p
hsa_circRNA_100451	RPS6KC1	−3.9851202	down	hsa-miR-200b-3p
hsa_circRNA_001283	WDR48	−3.8209534	down	hsa-miR-4668-3p
hsa_circRNA_103107	TPTE	−3.5046069	down	hsa-miR-203a-3p
hsa_circRNA_077409	MMS22L	−2.4438215	down	hsa-miR-141-3p
hsa_circRNA_102044	ACACA	−2.3354928	down	hsa-miR-205-5p
hsa_circRNA_104175	AMD1	−2.1360581	down	hsa-miR-223-3p
hsa_circRNA_101280	SLAIN1	−2.117463	down	hsa-miR-200c-3p

**Table 1C:** Two miRNA expressions significantly upregulated in wound of diabetic patients.

id	baseMean	baseMeanA	baseMeanB	foldChange	log2FoldChange	pval	padj
hsa-miR-223-3p	484.5559307	121.642098	847.4697635	6.966911765	2.800519292	0.041810423	1
hsa-miR-144-3p	1294.236145	338.9879054	2249.484385	6.635883905	2.730288647	0.046048015	1

**Table 1D:** Top 20 miRNAs significantly downregulated in wound of diabetic patients.

ID	baseMean	baseMeanA	baseMeanB	foldChange	log2FoldChange	pval	padj
hsa-miR-203a-3p	29,991.14994	59,954.34904	27.95084972	0.000466202	−11.06675655	5.3843E-06	0.0015682
hsa-miR-205-5p	37,980.28542	75,900.197	60.37383539	0.000795437	−10.29596446	2.42587E-05	0.0038147
hsa-miR-514a-3p	1579.89383	3151.961421	7.826237921	0.002482974	−8.653715192	1.84975E-06	0.0010124
hsa-miR-200c-3p	1976.907699	3942.635058	11.18033989	0.002835753	−8.462052319	2.60704E-06	0.0010124
hsa-miR-141-3p	477.9595302	952.5649584	3.354101966	0.003521127	−8.14974712	8.36984E-06	0.0019502
hsa-miR-184	129.3565325	257.595031	1.118033989	0.004340278	−7.847996907	9.91527E-05	0.0115513
hsa-miR-200b-3p	1926.819776	3829.042805	24.59674775	0.006423733	−7.282372407	1.84931E-05	0.0035908
hsa-miR-200a-3p	1259.577092	2499.029572	20.1246118	0.008052971	−6.956263209	2.92562E-05	0.0038147
hsa-miR-429	36.33610463	71.55417528	1.118033989	0.015625	−6	0.003674072	0.2351706
hsa-miR-187-3p	276.378002	543.8117321	8.94427191	0.016447368	−5.925999419	0.000318861	0.0285748
hsa-miR-200a-5p	332.9505218	654.7207038	11.18033989	0.017076503	−5.871843649	0.000304166	0.0285748
hsa-miR-200b-5p	22.47248317	43.82693236	1.118033989	0.025510204	−5.292781749	0.012306274	0.6516731
hsa-miR-141-5p	30.63413129	59.03219461	2.236067977	0.037878788	−4.722466024	0.012286719	0.6516731
hsa-miR-375-3p	266.3156961	508.0346445	24.59674775	0.048415493	−4.368387406	0.0038354	0.2351706
hsa-miR-224-3p	46.62201733	87.65386472	5.590169944	0.06377551	−3.970853654	0.017879493	0.8959104
hsa-miR-5571-3p	27.16822593	50.98234989	3.354101966	0.065789474	−3.925999419	0.028980729	1
hsa-miR-135b-5p	52.10038388	97.49256382	6.708203932	0.068807339	−3.861293729	0.018851041	0.8959104
hsa-miR-99a-3p	27.72724292	50.98234989	4.472135955	0.087719298	−3.510961919	0.04314336	1
hsa-miR-96-5p	178.1028144	323.7826431	32.42298567	0.100138122	−3.319936797	0.022611293	0.9407913
hsa-miR-1247-3p	45.61578674	82.28730157	8.94427191	0.108695652	−3.201633861	0.045270214	1

### Different expression profiles of circRNAs and miRNAs confirmed by qRT-PCR in normal tissues and diabetic wound

**Figure 2:**
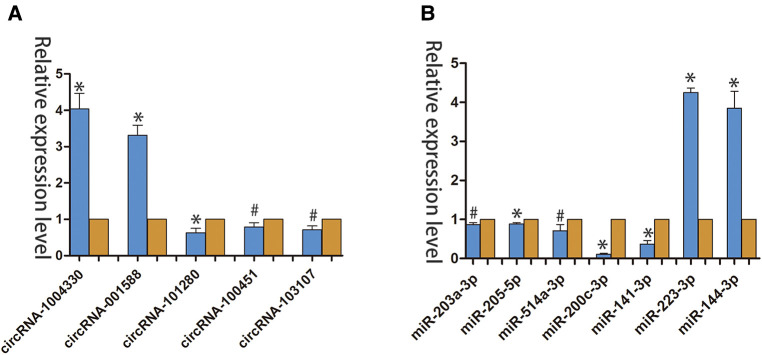
qRT-PCR verified the differential expression of circRNAs and miRNAs in Normal tissues and diabetic wounds. (**A**) Differential expression of circRNAs in different tissues. (*p < 0.01, #*p* < 0.05) (**B**) Differential expression of miRNAs in different tissues (*p < 0.01, #*p* < 0.05).(yellow indicates NC; blue indicates case.).

We used qRT-PCR to compared the expression profiles of circRNAs and miRNAs between normal tissues and diabetic wounds. The expression of circRNA-100451 and circRNA-103107 was consistent with our high-throughput gene sequencing results (Figure 2A). The large differences in RNA expressing were detected randomly. The expression levels of miRNA-223-3p and miRNA-144-3p were significantly upregulated in diabetic wounds, and those of miR-203a-3p, miR-205-5p, miR-514a-3p, miR-200c-3p, and miR-141-3p were significantly downregulated (Figure 2B).

### Go enrichment analysis of circRNAs and miRNAs expression in wound tissues of diabetic patient

**Figure 3:**
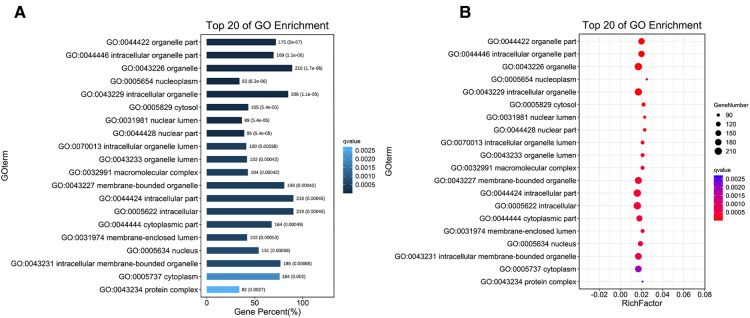
Go enrichment analysis of circRNA expression in wound tissues of diabetic patients. (**A**) The first 20 GO term entry columns with differential expression of circRNAs in diabetic wound tissues, GO with level 2 coordinates at the abscissa, and term enriched by each term. (**B**) GO enrichment analysis of differentially expressed cirRNAs in wound tissues of diabetic patients.

Three common gene databases, TargetScan, miRanda and mirbase, were used to predict target genes. It is considered as a potential regulatory gene of circRNA only when it is confirmed in the three databases. Then, go analysis was used to analyze the targeted function of the predicted target genes and pathway was used to analyze the signal pathway, so as to find the obvious enriched signal pathway that plays an important role in wound repair. Differentially expressed circRNA genes were analyzed by GO enrichment (Figures 3A–B). Based on these results, these differentially expressed CircRNA may be related to the GO functional annotations of biological processes, cellular components, and molecular functions (e. g., protein binding).

**Figure 4:**
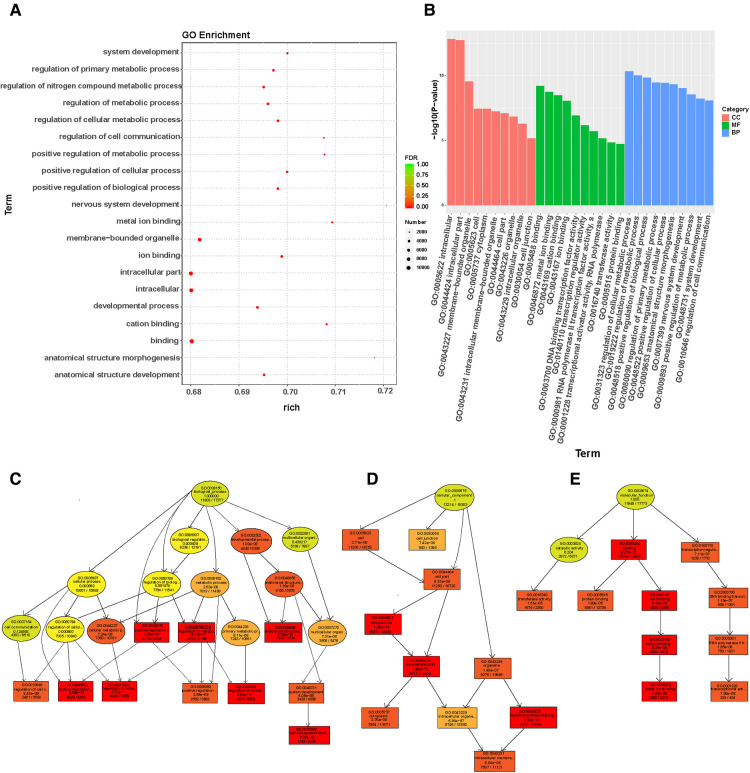
Go enrichment analysis of miRNAs expression in wound tissues of diabetic patients: (**A**) The first 20 GO term entry columns with differential expression of miRNAs in diabetic wound tissues, GO with level 2 coordinates at the abscissa, and term enriched by each term. (**B**) GO enrichment analysis of differentially expressed miRNAs in wound tissues of diabetic patients. (**C**) GO-BP map of the target genes in wound tissue of diabetic patients. (**D**) GO-CC map of the target genes in wound tissue of diabetic patients. (**E**) GO-MF map of the target genes in wound tissue of diabetic patients.

The target genes were used to predict among TargetScan, miRanda, and miRbase databases. Target genes were considered to be potential regulatory genes of miRNAs only when confirmed in the three databases. We performed GO enrichment analysis to analyze the function and signal pathways of predicted target genes. We found a significantly enriched signal pathway that may play an important role in wound repair. The cell component (CC), molecular function (MF), and biological process (BP) of differentially expressed miRNA target genes were classified by GO enrichment analysis. The upper 10 term entry with the smallest *p*-value. The most significant enrichment in each GO term was displayed (Figures 4A–B). The results of enrichment analysis provided directed acyclic maps of three GO ontologies. More important, GO term from colorless-light yellow to dark yellow-red according to the GO function analysis of the selected differentially expressed genes. The significant changes of BP were intercellular regulationmetabolic regulation, biological positive regulation, cell anatomical structure morphology, nervous system development, positive metabolic regulation, and cell to cell signal transduction regulation (Figure 4C). Significantly altered CC contain intercellular, intracellular, cytoplasmic, cell membrane binding site, organelles, and other cell components (Figure 4D). The important changes in MF were DNA binding transcription factor activity, intracellular metal ion binding, cation binding, intracellular transferase activity, protein binding, and RNA polymerase II transcription factor activity (Figure 4E).

### KEGG pathway enrichment analysis results of different genes

**Figure 5:**
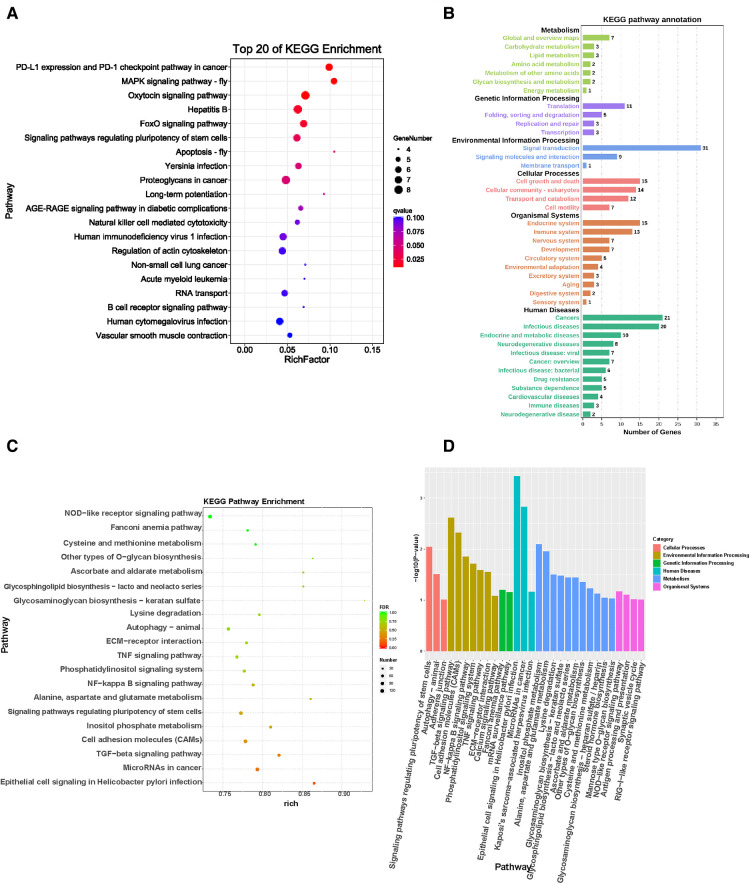
KEGG pathway enrichment analysis results of differential genes. (**A**) KEGG pathway enrichment results in differential expression of circRNAs in wound tissues of diabetic patients. (**B**). KEGG pathway enrichment analysis of differentially expressed circRNAs in diabetic wound tissues. (**C**) KEGG functional enrichment analysis of differentially expressed miRNAs in wound tissues of diabetic patients showed that the differentially expressed genes are involved in multiple signaling pathways. (**D**) KEGG pathway enrichment results of differentially expressed miRNA in diabetic wound tissues. According to the results of KEGG enrichment pathway analysis, the degree of enrichment was measured by rich factor, FDR values, and the number of miRNA target genes enriched on this pathway. The value range of FDR is 0–1. The closer it is to zero, the more significant the enrichment is. The top 20 KEGG pathways with the lowest FDR value and the most significant enrichment are selected for display. The abscissa is the name of the path, and the ordinate is—log10 (*p*-value) of each path.

According to KEGG pathway analysis, the host genes of these differentially expressed circRNAs were associated with acute myeloid leukemia (AML), vascular smooth muscle contraction, human cytomegalovirus infection, and the B cell receptor signaling pathway. Vascular smooth muscle contraction related pathways play an important role in the process of wound healing (Figure 5A). According to KEGG pathway analysis, the host genes of these differentially expressed circRNAs are mainly associated with infectious, endocrine, metabolic, and neurodegenerative diseases, as well as cancer. In cellular processes, circRNAs were mainly associated with cell value appreciation and apoptosis, cell community eukaryotes, and cell transport catabolism. circRNAs were also associated with signal transduction, including signaling molecules and molecule interaction, which is related to genetic information transformation processing. The differentially expressed circRNAs were involved in various metabolic functions (Figure 5B).

circRNAs can modulate miRNAs function by acting as a miRNAs sponge. Differential expression levels of miRNAs and parental gene transcripts were studied to determine their biological effects. Compared with normal skin tissues, KEGG analysis showed that miRNAs were overexpressed in diabetic wounds and were associated with ubiquitin like protein synthesis and receptor activity of nuclear output signals (Figure 5C). In addition, KEGG pathway analysis revealed that dysregulated miRNAs were associated with vascular smooth muscle contraction related pathways and regulation of the actin cytoskeleton (Figure 5D). Based on these data, the biological functions of these miRNAs may contribute to the study of chronic refractory wounds in diabetes.

### Construction of CeRNA network of miRNAs and cirRNAs

**Figure 6:**
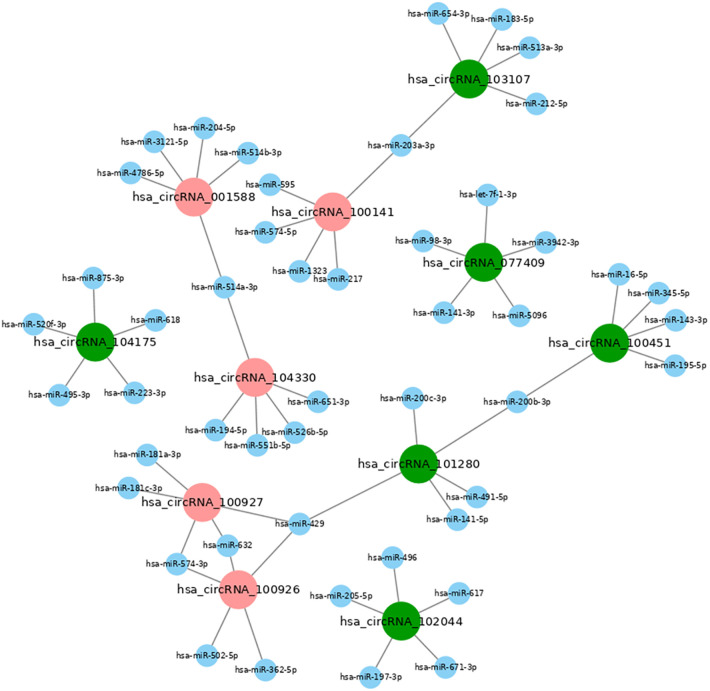
CeRNA network map of differentially expressed miRNAs and circRNAs in diabetic wound tissues. The ceRNA network is based on circRNA/miRNA and mRNA/miRNA interactions. In this network, circRNA expression is associated with mRNAs through miRNAs. The results showed that miR-223-5p and miR-144-5p are up-regulated in diabetic patients with chronic refractory wounds; hsa_circRNA_104175 is down-regulated and is related to miR-223-5p. miR-203-3p, miR-205-5p, miR-514a-3p, miR-200c-3p and miR-141-3p are down-regulated. Hsa_circRNA_100141 is up-regulated, hsa_circRNA_ 103107 is down-regulated, and both are related to the regulation of mir-203-3p. Hsa_circRNA_ 102044, hsa_circRNA_101280, hsa_ circRNA_077409 are down-regulated and related to the regulation of miR-514a-3p, miR-200c-3p, and miR-141-3p; Hsa_circRNA_001588 and hsa_circRNA_104330 are upregulated and are related to miR-514a-3p regulation; Hsa_circRNA_100451 and hsa_circRNA_101280 are downregulated and related to miR-200b-3p regulation; Hsa_circRNA_100926 and hsa_ circRNA_100927 are up-regulated, Hsa_ circRNA_ 101280 is down-regulated, and both are related to miR-429 regulation.

**Figure 7:**
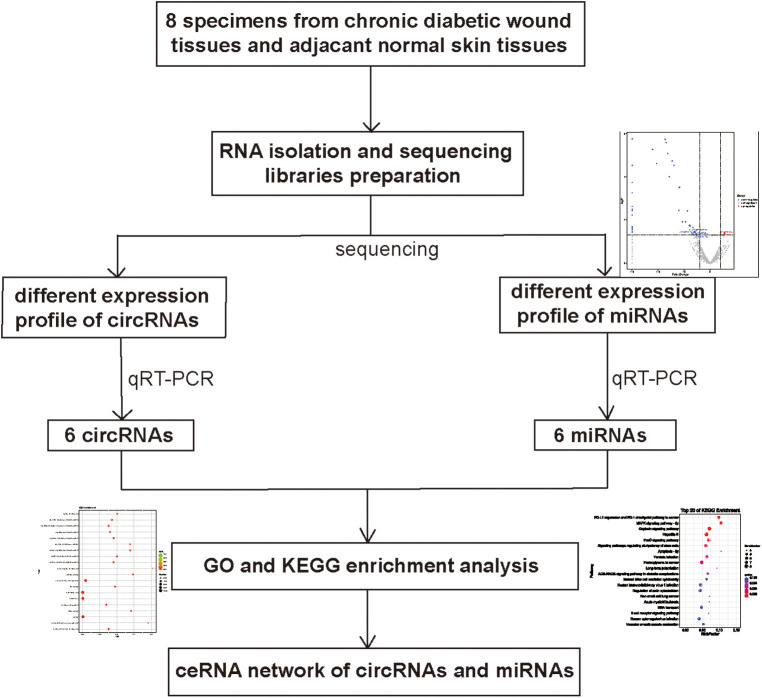
Flowchart for circRNA-miRNA network construction.

The prediction of function of circRNAs relied on the annotation of co-expressed miRNAs to obtain core circRNAs which were associated with wound healing. We selected significantly related gene pairs to construct co-expression etworks encoding noncoding genes, and determined the interaction and importance of circRNAs and miRNAs. This finding facilitated the search of modulation between circRNAs and miRNAs associated with diabetic wounds. The 11 circRNAs and 24 miRNAs which were differentially expressed were selected. Many circRNAs and miRNAs can act as ceRNA. They compete with each other for the same miRNAs and adjust each other. ceRNA networks were established using high-throughput sequencing data in our study (Figure 6). A flow chart of the analysis done shown in Figure 7.

## Discussion

CircRNAs are involved in both post-transcriptional and transcriptional gene expression regulation in previous reports. circRNAs act as miRNA sponges and are enriched with functional miRNA binding sites. The disturbed circRNAs may play an important role in the occurrence and development of diabetes (18). For example, Xu et al. (19) found that the circRNA Cdr1as (aka ciRS-7) and miR-7 were expressed in islet cells in *in vitro* cell experiments. Cdr1as acts as an RNA sponge for miR-7 and suppresses its activity in islet cells by binding miR-7, thereby increasing insulin release and synthesis from the translational and transcriptional levels. This study demonstrated the sponge adsorption of Cdr1as on miR-7 and demonstrated the regulation of Cdr1as and miR-7 on insulin synthesis (20). Release from the transcriptional and translational levels in islet cells suggested that they may be important regulators in the development of diabetes. These data indicate that ncRNA expression is significantly altered *in vitro* in a diabetes model, which may also be related to the development of diabetes. However, the expression profiles of circRNAs and miRNAs and their associated co-expression have rarely been reported in studies on diabetic chronic refractory wounds. There is also sparse literature related to ceRNA networks in this field.

In our study, the expression profiles of circRNAs and miRNAs in chronic refractory wounds and normal skin of diabetic patients were detected by RNA-seq. Coding noncoding gene co-expression and ceRNA networks were constructed using these expression profiles. RNA-seq analysis indicated that the expression levels of 356 circRNAs and 42 miRNAs varied between the study groups. Six circRNAs and miRNAs with significant differences in expression and high correlation where selected and verified by qRT-PCR to confirm the reliability of the results. The differentially expressed circRNAs may be related to the GO functional annotation of BP, MF (such as protein binding), and CC according to the GO enrichment analysis of the above differentially expressed miRNAs and circRNAs. The differentially expressed miRNAs are mainly involved in the metabolic regulation of intracellular and cell membrane substances, the positive regulation of body metabolism, the remodeling of anatomical structure and the regulation of morphological development. KEGG enrichment analysis showed that the differentially expressed circRNAs host genes are mainly related to acute myeloid leukemia, B cell receptor signaling, RNA transport, vascular smooth muscle contraction related pathways, and human cytomegalovirus infection. This data suggest that circRNAs may be involved in the regulation of diabetic chronic refractory wound healing process. These circRNAs may play an important role in the vascular smooth muscle contraction related pathways in the wound healing process. According to KEGG enrichment analysis, the host genes of these differentially expressed circRNAs are mainly related to infectious, endocrine, metabolic, and neurodegenerative diseases, as well as cancer (21). In the body system, it has high correlation with the endocrine system, immune system (22), nervous system (23) and development. In cell process mechanisms, it is significantly related to cell proliferation and apoptosis (24), eukaryotes of cell community, and cell transportation and catabolism (25). In environmental information processing, circRNAs were related to signal transduction and interaction between signal molecules *via* transformation of genetic information processing and distribution in metabolism. Differentially expressed miRNAs in our study were mainly involved in the TNF signaling pathway, the NOD-like receptor signaling pathway, the Fanconi anemia pathway, methionine metabolism and cysteine, autophagy, ECM-receptor interaction, phosphatidylinositol signaling, NF-kappa B signaling pathway and the TGF-beta signaling pathway. KEGG pathway analysis indicated that the host genes of these differentially expressed miRNAs are closely linked to ECM components, including B cell receptor signaling, RNA transport, and TGF signaling. These genes may play important roles in apoptosis, metastasis, differentiation, and cell proliferation. These differentially expressed miRNAs were related to new tissue formation, remodeling, inflammation, and trauma repair.

The mechanism of ceRNA suggests that RNA molecules carrying the MRE communicate with each other by competing for the same miRNA sites. This new understanding of RNA crosstalk can provide a different explanation of the function of non-characteristic ncRNAs in various diseases (26). Prediction of the ceRNA crosstalk depends on the identification of the MRE on the relevant transcripts. ceRNA was found to participate in various biological processes in the previous studies. The biological processes are associated with various diseases, including endocrine and metabolic-related diseases (27, 28). However, there is no research about the function of the RNA crosstalk in the chronic refractory wounds of diabetes. A ceRNA network of chronic refractory diabetic wounds was constructed based on RNA sequence data in this study. The network showed miRNA-circRNA mRNA correlation in chronic refractory diabetic wounds, including 11 circRNAs and 24 miRNAs. There were significant differences in the expression levels of hsa _ circRNA_ 104175, hsa_ circRNA_ 001588, hsa_ circRNA_ 104330, hsa_ circRNA_ 100141, hsa_ circRNA_ 103107, and hsa_ circRNA_ 102044, which has a network communication relationship with miR-205-5p and miR-223-5p. Studies have found that circ_0001588 acts as a ceRNA and promotes HCC progression by targeting the miR-874/CDK4 signaling pathway, circ_0001588 may be a promising target for HCC treatment (29). The expression of circ_0001588 was markedly up-regulated in glioblastoma tissues and human gliomas cells, moreover, circ_0001588 accelerated the proliferation, migration and invasion of glioblastoma by modulating miR-211-5p/YY1 signaling (30). Wang et al. (31) indicated that circ_0001588 acted as an oncogene in glioma malignant progression by miR-1281/ERBB4 pathway, suggesting the potential of circ_0001588 as a therapeutic target for glioma. Another study revealed that hsa_circ_0001588 upregulated the expression of NACC1 by combining with miR-524-3p to promote the proliferation, migration, and invasion of lung adenocarcinoma cells (32). Chen et al. (33) suggested that hsa_circ_0007364 might serve as an oncogenic circRNA in CC progression by regulating the miR-101-5p/MAT2A axis. However, hsa_cicr_104175, hsa_circ_104330, hsa_circ_10307, hsa_circ_102044 were not reported in other studies.

Although the action mechanism of circRNAs in diabetes has not been reported in literature, the role of miRNA in diabetes mellitus has been studied. For example, miR-223-5p, which has a network communication relationship with hsa_circRNA_104175, was significantly lower in megakaryocytes treated with high glucose. This suggests that miR-223-5p can negatively regulate platelet formation and affect platelet morphology and function in diabetic hyperglycemia, thereby accelerating atherosclerosis in peripheral tissues and promoting arterial thrombosis (34). The miR-223-5p of bone marrow derived can regulate the apoptosis and proliferation of vascular endothelial cells, that realize through NOD-like receptor signaling pathway of vascular endothelial cells and participate in the process of vascular injury in vascular related diseases (35). The vascular endothelial cells highly express miR-223-5p that directly leads to an increase in apoptosis. The increasing apoptosis leads to coronary artery injury, vascular thrombosis, and coronary aneurysm of cardiovascular system. MiR-205-5p and miR-514a-3p have a network communication relationship with the circRNAs including hsa_circRNA_001588, hsa_circRNA_104330, and hsa_circRNA_102044. The downregulated miR-205-5p and miR-514a-3p regulate the expression of vascular endothelial growth factor A and regulate E2F1 and Bcl-2 through tyrosine kinase signal transduction pathway. miR-205-5p and miR-514a-3p also can regulate the local tissue cell activity through the TGF β signaling pathway (36). hsa_circRNA_100141 and hsa_circRNA_103107 have a reticular communication relationship with miR-203a-3p. miR-203a-3p can activate and regulate the apoptosis of different types of cells through the action of its target gene, MEKK1, and the phosphatidylinositol signaling system, ERK1/2, P38, and JNK. The downregulated miR-203a-3p can accelerate the apoptosis of wound tissue cells through the action of MEKK1 (37).

These results showed that the related circRNAs and miRNAs are involved in the regulation process of healing of chronic refractory wounds in diabetes. The related circRNAs and miRNAs play important role in the ceRNA network of chronic refractory wounds in diabetes. Therefore, although the specific role of these circRNAs and miRNAs in the chronic refractory diabetic wounds is still unclear, the regulatory network analysis indicates that it may be highly related to the chronic refractory diabetic wound healing mechanism. In future studies, we will continue to collect tissue samples from chronic refractory wounds of diabetes and verify the relationship between these miRNAs and circRNAs *in vitro*, identify key genes and pathways related to diabetic chronic refractory wound healing, and elucidate their relationship.

RNA-seq can help elucidate the key role of ncRNAs in the occurrence and development of diseases, although the functions of most miRNAs and circRNAs are still unclear. In this study, many ncRNAs, including miRNAs and circRNAs, were found to be differentially expressed in diabetic chronic refractory wounds compared with normal skin tissues. However, because this study is a single center study and the sample size is small, we will further explore the related genes and pathways from a large sample from multiple centers in future studies.

## Conclusions

Our study showed that a specific ceRNA network is closely related to the occurrence and development of diabetes refractory wounds, which may help to identify molecular biomarkers and therapeutic targets for diabetes refractory wounds. Although these results may need further validation, these differentially expressed miRNAs, circRNAs, and ceRNA network are helpful to explore the pathogenesis of diabetes refractory wounds and find clinical biomarkers related to their treatment. We will also perform further in-depth research in follow-up studies to provide new strategies for the treatment of chronic refractory wounds of diabetes.

## Data Availability

The original contributions presented in the study are publicly available. This data can be found here: GEO GSE212167.
